# Tacrolimus Concentration/Dose Ratio: A Tool for Guiding Tacrolimus Dosage Post-renal Transplantation

**DOI:** 10.7759/cureus.53421

**Published:** 2024-02-01

**Authors:** Mamatha T Shenoy, Jeyakumar Manavalan, Hariharan A, Suganthy K, Pradipta Kumar Mohanty

**Affiliations:** 1 Biochemistry, Velammal Medical College Hospital and Research Institute, Madurai, IND; 2 Biochemistry, Sri Manakulavinayagar Medical College and Hospital, Puducherry, IND

**Keywords:** concentration/ dose ratio, cyp3a5 genotype, nephrotoxicity, post-renal transplant, tacrolimus

## Abstract

Background

The calcineurin inhibitor, Tacrolimus (Tac), exhibits variable absorption and undergoes first-pass metabolism when administered orally. The narrow therapeutic window and individual variability of this immunosuppressive agent make therapeutic drug monitoring essential. We hypothesized that the Tac metabolism rate - defined as the blood concentration normalized by its daily dose (the C/D ratio) - is associated with post-renal transplant (RTx) function.

Methodology

A retrospective observational study was conducted including 40 RTx patients. Clinical reports from four follow-up ambulatory appointments at one, three, six, and 12 months were analyzed. Tac dose and its blood levels were used to calculate the Tac concentration/dose (C/D) ratio. Patients with a Tac C/D ratio <1.05 ng/mL x 1/mg and a C/D ratio >1.05 ng/mL x 1/mg were categorized as fast and slow metabolizers. Serum creatinine levels were compared between the two groups, and their association with the Tac C/D ratio was analyzed. Student's unpaired t-test and the Mann-Whitney U test were used to analyze the difference in the C/D ratio between the groups. Spearman correlation analysis was conducted to analyze the association of the C/D ratio with serum creatinine in both groups. A *P*-value of <0.05 was considered statistically significant.

Results

Fast metabolizers showed increased serum creatinine (*P* < 0.05), and the C/D ratio correlated with creatinine levels. ROC analysis used to identify fast metabolizers for the C/D ratio at three months had an area of 0.925 (*P* < 0.01).

Conclusions

The Tac C/D ratio can be used as an earlier diagnostic tool to predict the development of nephrotoxicity in RTx patients.

## Introduction

Tacrolimus (Tac) is one of the most effective immunosuppressive drugs used in post-renal transplant (RTx) to prevent organ rejection. It is used in combination with mycophenolate mofetil and prednisolone [1]. Because of its lipophilic nature, absorption of oral Tac varies between individuals, which can influence its efficacy and toxicity [2]. The narrow pharmaceutical window and high inter-individual variation of Tac greatly influence the pharmacokinetics of the drug, and it necessitates the frequent monitoring of blood Tac levels and dose adjustments when needed [3]. It is essential to maintain the optimal levels of Tac to prevent acute or chronic graft rejection. Lower Tac trough level was found to be associated with acute graft rejection within one year of renal transplant [4]. On the other hand, high trough levels of Tac were associated with delayed graft rejection, nephrotoxicity, neurotoxicity, and diabetogenic effects [5]. Calcineurin inhibitor (CNI)-related nephrotoxicity (CNIT) can be either acute or chronic. Moreover, nephrotoxicity occurs not only with higher troughs of Tacrolimus but also with lower trough concentrations. It is very crucial to identify the patients at risk for developing such nephrotoxicity at the earliest. Although nephrotoxicity is attributed to the Tac concentration [6], its metabolism also plays a major role in it. Apart from its absorption, various factors like albumin levels in the blood, old age, and male gender influence the metabolism of Tac [7]. Also, the drug interaction of Tac with other immunosuppressive agents makes an impact while optimizing the daily dosage of Tac [8]. The inter-individual variation of Tac can be attributed to polymorphisms in the gene encoding the cytochrome P450 (CYP) enzymes CYP3A4 and CYP3A5 [9]. However, gene polymorphic studies on RTx patients and optimizing the dosage of Tac may not be possible in routine, and there is a need for a simple measurement tool that could predict the nephrotoxic effects. Recently, it was observed that the Tac metabolism rate, expressed as the blood concentration of Tac normalized by Tac dose (C/D ratio), has an impact on kidney function after RTx [7]. The Tac C/D ratio can be used to establish the Tac rate of metabolism. Serum creatinine is a well-established parameter to assess the renal impairment. Even a slight elevation in serum creatinine from the baseline values has got its significance. In this study, we aimed to test our hypothesis that the Tac metabolism rate, expressed as the C/D ratio, is associated with renal function in RTx recipients.

## Materials and methods

We conducted a hospital-based retrospective observational study in the Department of Biochemistry after approval from the Institutional Ethics Committee. We adhered to all ethical principles for medical research involving human subjects, following the Declaration of Helsinki of 1975, revised in 1983. Reports based on Tac data indicated that all patients who underwent renal transplants between 2017 and 2020 and attended the center’s outpatient post-transplantation clinics were screened. We analyzed clinical reports from four follow-up ambulatory appointments post-transplantation in the first, third, sixth, and 12th months. A standard formulation of Tac (Prograf) was administered to patients at our institute. Renal function was evaluated at each visit using serum creatinine. A daily dose of Tac received before blood withdrawal was recorded for all patients. From the available data of Tac dosage and blood Tac levels, Tac metabolism rates were calculated by normalizing the Tac blood trough concentration (C) with its corresponding daily Tac dose (D) using the formula [10,11]:

C/D ratio (ng/mL X 1/mg) = Blood Tac trough concentration (ng/mL)/Daily Tac dose (mg)

Very high Tac trough concentrations (>15 ng/mL) were not taken into consideration as they can be false-high values followed by Tac intake immediately before blood sampling.

Inclusion criteria

Patients who were compliant with Tac medication in the form of tablets for at least one year post-transplantation were included as the study subjects.

Exclusion criteria

Patients who were non-compliant with the medication, those lost to follow-up, and those who had been switched to alternate immunosuppressive therapy were excluded from the study.

Based on the Tac C/D ratio, patients with a C/D ratio <1.05 ng/mL x 1/mg were defined as fast metabolizers and patients with a C/D ratio of >1.05 ng/mL x 1/mg were defined as slow metabolizers [11].

Serum creatinine was analyzed and compared in both groups, and its association with the Tac C/D ratio was examined. The quantitative estimation of blood Tac levels was assessed through immunoturbidimetric analysis using a TBA 120FR fully automated clinical chemistry analyzer. Serum creatinine was estimated using the enzymatic creatinine method. During analysis, laboratory standard operating procedures were followed with internal quality control materials.

Statistical analysis

The continuous variables with non-normal distribution using Kolmogorov-Smirnov's test were expressed as the median (25th percentile, 75th percentile). Data that were continuous and equally distributed were reported as mean with standard deviation. The categorical variables were summarized as frequencies and percentages. Student's unpaired t-test and Mann-Whitney U test were used to calculate the differences between the two metabolism groups (fast and slow). A *P*-value < 0.05 was considered statistically significant. The association of the C/D ratio with serum creatinine of the patients in both groups was assessed using Spearman correlation analysis. Receiver operating characteristic (ROC) curve analysis was conducted to determine the utility of the Tac C/D ratio in differentiating between fast metabolizers and slow metabolizers. Data were analyzed using SPSS for Windows, Version 16.0 (SPSS Inc., Chicago, IL) software.

## Results

In total, 40 renal transplant patients, consisting of 10 (25%) females and 30 (75%) males, fulfilled the inclusion criteria of having good compliance with the immunosuppressive regimen were included in the study. Their mean age was 38.12 ± 11.7 years at the time of renal transplantation. Based on the C/D ratio, the RTx patients were categorized into fast and slow metabolizers. Out of 40 patients, 13 were fast metabolizers and 27 were slow metabolizers. The Tac dose was compared between the two groups. The Tac dose was low in slow metabolizers throughout the study, and it was significantly lower in the third and sixth months (Table 1).

**Table 1:** Comparison of the Tacrolimus dose between fast and slow metabolizers presented as mean ± standard deviation (SD). An unpaired t-test was used to compare between the groups, and *P*-values < 0.05 were considered statistically significant.

Time after transplant	Fast metabolizers (mg)	Slow metabolizers (mg)	*P*-value
Post-op day 4	5.68 ± 2.95	4.2 ± 1.71	0.66
One month	10 ± 3.01	5.01 ± 2.9	0.184
Three months	7.8 ± 2.56	3.65 ± 1.7	0.017*
Six months	6.09 ± 3.15	2.5 ± 1.17	0.002*
12 months	5.31 ± 2.15	3.2 ± 3.25	0.47

The Tac C/D ratio was high in slow metabolizers compared to fast metabolizers throughout the study, and it was significantly higher in the sixth month (Table 2).

**Table 2:** Comparison of the Tacrolimus C/D ratio (ng/mL x 1/mg) between fast and slow metabolizers presented as mean ± standard deviation (SD). An unpaired t-test was used to compare between the groups, and *P*-values < 0.05 were considered statistically significant.

Time after transplant	Fast metabolizers	Slow metabolizers	*P*-value
Post-op day 4	1.0 ± 0.7	2.67 ± 2.5	0.32
One month	0.83 ± 0.27	2.45 ± 1.8	0.28
Three months	1.13 ± 0.42	2.76 ± 1.5	0.24
Six months	1.37 ± 0.9	3.3 ± 1.4	0.02*
12 months	1.5 ± 0.44	3.31 ± 1.8	0.20

Serum creatinine levels were found to be increased in fast metabolizers, with a statistically significant rise observed during the first and sixth months RTx (Table 3).

**Table 3:** Comparison of serum creatinine levels (mg/dL) between fast and slow metabolizers presented as median (25th quartile, 75th quartile). Mann-Whitney U test was used to compare between the groups, and *P*-values < 0.05 were considered statistically significant.

Time after transplant	Fast metabolizers, median (25th quartile, 75th quartile)	Slow metabolizers, median (25th quartile, 75th quartile)	*P*-value
Post-op day 4	1.8 (1.6, 3.9)	1.2 (0.9, 2.0)	0.07
One month	1.2 (1.1, 1.9)	1 (0.7, 1.3)	0.04*
Three months	1.1 (1, 1.15)	0.9 (0.75, 1.05)	0.15
Six months	1.05 (1, 1.18)	0.8 (0.55, 0.9)	0.02*
12 months	1.35 (1.08, 1.7)	0.8 (0.68, 1.03)	0.05

**Figure 1:**
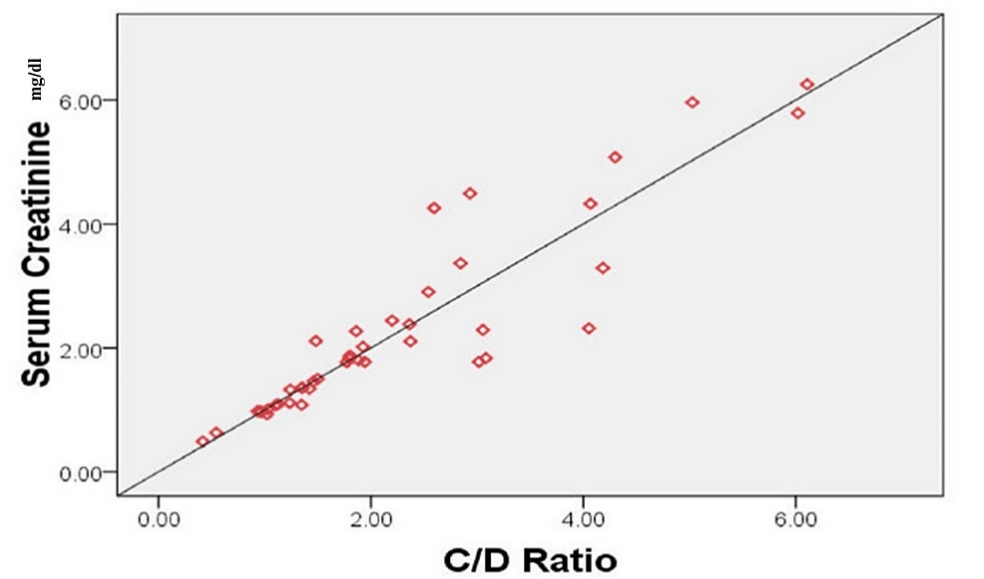
Correlation of serum creatinine levels with the Tac C/D ratio. Spearman correlation between serum creatinine levels (mg/dL) and the Tac C/D ratio; *r*-value = 0.935, with a *P*-value of <0.001. Tac, Tacrolimus; C/D, concentration/dose

A significant correlation was observed between serum creatinine levels and the Tac C/D ratio (Figure 1).

**Figure 2:**
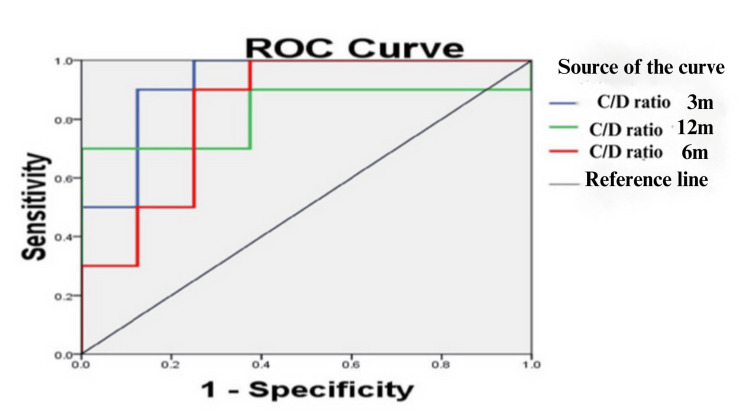
ROC curve analysis: Comparison of the ability of the C/D ratio (Tac trough concentration normalized by the Tac daily dose) to identify fast metabolizers among renal transplant recipients at three, six, and 12 months, respectively. Tac, Tacrolimus; C/D, concentration/dose; ROC, receiver operating characteristic

ROC curve analysis was performed to predict the ability of the C/D ratio to identify fast metabolizers among renal transplant recipients (Figure 2). The areas under the curve at three, six, and 12 months were 0.925 (95% confidence interval [CI] 0.794-1.096), 0.838 (95% CI 0.635-1.040), and 0.825 (95% CI 0.616-1.034), respectively (Table 4).

**Table 4:** Area under curve of ROC analysis. ROC curve analysis to analyze the ability of the C/D ratio to identify fast metabolizers. C/D, concentration/dose; ROC, receiver operating characteristic; CI, confidence interval

Test variable	Area under ROC	Significance	95% CI
C/D ratio three months	0.925	0.003*	0.794-1.096
C/D ratio six months	0.838	0.016*	0.635-1.040
C/D ratio 12 months	0.825	0.021*	0.616-1.034

## Discussion

Tac, a powerful immunosuppressive agent, is macrolide by nature. It is the most preferred CNI in renal transplant patients, whether pediatric or adult, to prevent allograft rejection. The major challenge lies in the pharmacokinetics of Tacrolimus; high blood trough levels can predispose to nephrotoxicity, and low levels may be associated with reduced immunosuppression, leading to graft rejection. Hence, the right balance must be maintained between efficacy and toxicity. In this retrospective study, we analyzed the C/D ratio of Tac in renal transplant recipients and its association with the renal function expressed as serum creatinine levels. We observed that the Tac C/D ratio was significantly lower in fast metabolizers and the daily dosage of Tac was higher in fast metabolizers, which can be explained by the fact that clinicians must increase the dose of Tac to achieve the adequate target Tac trough concentration. Hence, fast metabolizers tend to be overexposed to immunosuppression in the earlier phases after renal transplant [12]. The slow metabolizers needed lower Tac dosage to attain the required blood concentration to produce the necessary immunosuppression (Table 1).

Egeland et al. observed that RTx patients with high Tac clearance developed graft interstitial fibrosis and tubular atrophy (IFTA) as early as seven weeks after renal transplant. Additionally, patients requiring a high Tac dosage, up to 7.5 mg per day, were found to be twice as likely to develop IFTA compared to patients taking a low Tac dosage of about 2.5 mg per day [13]. Tac metabolism was affected by numerous biological variants, including age, body mass index, hematocrit, serum albumin levels, and gender of the transplant recipient [1,14]. The prominent variable that affected the metabolism of the drug was the CYP3A4 and CYP3A5 enzyme-encoding genes. Tac was metabolized by cytochrome P450 (CYP) 3A5, expressed in the cells of the human liver and intestine. Both CYP3A4 and CYP3A5 played a role in the metabolism of Tac, whereas CYP3A5 genes caused variations in bioavailability amongst individuals [15]. The literature review confirmed that the mutation of (G6986A) produced the CYP3A5*3 allele. Non-expressers of CYP3A5*3 were found to lose their functional enzyme, and the expression of CYP3A5*1 led to higher enzymatic activity. CYP3A5*1 or CYP3A5*3 could be a key factor in the pharmacokinetics of Tac. The dose-modifying effect of the single gene polymorphism in CYP3A5 was seen to be more predominant in the Asian population than in Western individuals [16]. Individuals who are homozygous for the polymorphism in the allele (CYP3A5*3/*3) are deficient in the production of the enzyme and are termed non-expressers. Expressions may be homozygous (CYP3A5*1/*1) or heterozygous (CYP3A5*1/*3) in nature, irrespective of which they will increase the metabolism of Tac due to the production of the metabolizing enzyme. In a study conducted by Zheng et al., expressers achieved a twofold lower Tac C/D ratio compared with non-expressers [17]. Our study population supported the finding of a higher C/D ratio in slow metabolizers (Table 2). However, the limitations of genetic testing are its availability in routine settings and its cost. We opine that a simple and cost-free calculation is preferred to genetic testing, which can be an additional burden to the already-stressed patients.

Ethnicity could influence Tac dosage, as the metabolism of Tac is affected, leading to variation in dosage [18]. Persons of African origin may exhibit a higher Tac dosage requirement due to differences in CYP3A compared to individuals of Asian or Caucasian descent [19]. Such racial variations have been avoided in our study by considering an ethnically homogenous study population.

Kwiatkowska et al. and Nowicka et al. reported a decline in renal function in fast metabolizers [19,20]. The renal function, as measured by serum creatinine values in our population, was lower in slow metabolizers, as their blood Tac levels were better maintained to provide the required immunosuppression. We also observed serum creatinine was elevated in fast metabolizers throughout the study period. The C/D ratio showed a strong correlation with renal function in the study cohort. The underlying mechanism for Tac nephrotoxicity remains unclear still. van Gelder et al. proposed that fast metabolizers may have higher concentrations of Tac metabolites in the blood, which may accumulate in the renal tubules, leading to nephrotoxicity [21]. Moreover, high Tac clearance may impact fast metabolizers in achieving the required trough levels and could result in under-immunosuppression, at least at some time of the day. This could account for additional injury to the graft's health.

Limitations of this study

Limitations of this study include its retrospective nature and reliance on document analysis. Additionally, the genetic polymorphisms of the various CYP3A5 enzymes have not been individually studied.

Implications of this study

The distribution of patients based on the C/D ratio helps in analyzing the type of metabolism and determining the predominance of either slow or fast metabolizers within the ethnic group. This knowledge helps to regulate and monitor Tac dosage in patients after RTx.

The association of the C/D ratio with renal function helps in detecting nephrotoxicity earlier and guides any modifications to the treatment when required.

## Conclusions

This study concludes on the utility of the Tac C/D ratio in all patients receiving Tac after renal graft transplantation. Tac C/D can mirror the genetic mutations of these enzymes that may interfere with the metabolism and pharmacokinetics of Tac, leading to the expression of poor or fast metabolizer proteins. This can provide a very useful tool for clinicians to monitor graft status in RTx patients.
